# Supplementary material for: Persuasive Messages Will Not Increase COVID-19 Vaccine Acceptance: Evidence from a Nationwide Online Experiment
Source: Vaccines (Basel). 2021 Sep 30;9(10):1113. doi: 10.3390/vaccines9101113 (PMC8539857; doi:10.3390/vaccines9101113)
Supplement: Supplementary file 1 [file vaccines-09-01113-s001.zip › vaccines-1370667-supplementary.pdf]

Supplementary Information for
Persuasive messages will not increase COVID-19 vaccine acceptance: Evidence from a nationwide online experiment

Raman Kachurka, Michał Krawczyk*, Joanna Rachubik
Faculty of Economic Sciences, University of Warsaw, Warsaw, Poland.
Email: mkrawczyk@wne.uw.edu.pl

This file includes:

Supplementary S1– Questionnaire (both waves)
Supplementary S2 – Overview: categorization of open-ended questions
Supplementary S3 – Procedure: categorization of open-ended questions
Supplementary S4 – Detailed results: categorization of open-ended questions
Supplementary S5 – Additional tables for Wave 1
Supplementary S6 – Additional tables for Wave 2
Supplementary S7 – Summary of demographic characteristics, Wave 1 and Wave 2
Figures S1.1 to S3.2
Tables – Table S1.1 to Table S5.1

Supplementary S1– Questionnaire (both waves)

[text in square brackets was not visible to the subjects]

We invite you to fill in the questionnaire as part of the research conducted for the University of Warsaw. Please answer according to your beliefs. We guarantee full confidentiality of the collected data.

The survey will take you about 15 minutes to complete, and you will receive [xx] Ariadna points for it.

The survey consists of several different thematic blocks. Please complete the survey at once and do not take breaks. While completing the survey, it is not possible to return to the previous page.

To participate in the survey, please continue.

Greetings

In case of any problems with the survey, or if you would like to provide us with your comments on the survey, please contact us:

[sex] What is your gender?

[rotate]
female
male

[age] What age are you?

18-24 years old
25-34 years old
35-44 years old
45-54 years old
55 years old or older

[year] What year were you born?

[ ]

[city_population] What is the size of the community you live in?

village
small town (up to 20 000 inhabitants)
medium city (between 20 000 and 99 000 inhabitants)
large city (between 100 000 and 500 000 inhabitants)
large city (more than 500 000 inhabitants)

[woj] What voivodeship do you live in?

Dolnośląskie
Kujawsko-Pomorskie
Lubelskie
Lubuskie
Łódzkie
Małopolskie
Mazowieckie
Opolskie
Podkarpackie
Podlaskie
Pomorskie
Śląskie
Świętokrzyskie

Warmińsko-Mazurskie
Wielkopolskie
Zachodniopomorskie

[edu] What is your current education (the last school completed)?

Primary or lower secondary school
elementary
secondary
post-secondary
currently studying
bachelor’s degree
higher education completed

[if in edu: bachelor or higher education]
[M7] What was your field of study?

[in if edu: currently studying]
[M7a] What is your field of study?

[P17] How strongly are you experiencing the following emotion at the moment?

[horizontal scale: 1 I do not feel this emotion now, 10 I feel this emotion very strongly right now]

[rotate and register order]
Joy
Fear
Anger
Repulsion
Sadness
Surprise

[only in wave 2]
[trust_gov, trust_neighbours, trust_doctors, trust_media, trust_family, trust_scientists] Do you trust:

[rotate and register order]
representatives of the European Union
the government
your neighbors
doctors
the media (journalists)
your family
scientists

[scale:] YES, high
YES, moderate
NO
I have no opinion

[P18] How do you perceive your willingness to take risks in general?

[horizontal scale: 1 totally risk-averse, 10 totally willing to take risks]

[P19] People may behave differently in different situations. How would you rate your willingness to take risks in the following cases?

[horizontal scale: 1 totally risk-averse, 10 totally willing to take risks]
[rotate and register order]
Your professional work

Taking care of your health, such as undergoing surgery that may bring complications, putting off recommended medical tests, changing a prescribed medication without consulting your doctor, having unprotected sex with someone other than a long-term, faithful partner.

[P37] Read the description below and then answer the related questions.

A vaccine for coronavirus has recently become available in Poland. Vaccination is voluntary.

[*Randomized messages – each could be present or not. Exactly three randomly selected messages were shown in Wave 1: each message was independently drawn or not (50/50) in Wave 2*]
[*v_producer_reputation***]**
The vaccine was developed by scientists from the American Pfizer and the German Biontech. [wave 1]/ The vaccine was developed by scientists from an international research consortium. [wave 2]

[*v_efficiency***]** The vaccine's effectiveness has been estimated at over 90%, which means that a vaccinated person is more than ten times less likely to get the disease than an unvaccinated person.

[*v_safety***]** The European Medicines Agency confirms that the vaccine is safe. Possible side effects are mild to moderate, can be treated with paracetamol, and disappear within a few days.

[v_other_want_it**]** Research conducted by IPSOS on 18 000 people in 15 countries shows that about 75% want to get vaccinated as soon as possible.

[*v_scientific_authority***]** According to the COVID team at the Polish Academy of Sciences, "vaccination is the only rational choice, thanks to which we will be able to exit the pandemic faster." The use of the vaccine is also recommended by the Supreme Medical Chamber and many other medical and scientific societies.

[*v_ease_personal_restrictions***]** It should be assumed that vaccination will make everyday life easier: vaccinated people will not have to quarantine after contact with an infected person, will be able to travel freely abroad, will not have to wear a face mask, etc.

[*v_scarcity***]** *[only in wave 1]* In the initial stages, there will not be enough vaccines for everyone.

[*v_tested***]** [only in wave 2] Development work on the vaccines began immediately after the pandemic outbreak and was treated as a priority. It drew on the vast experience of the research teams involved and used some of the solutions that had been used in vaccines for years. In total, more than 100 000 people were tested in clinical trials.

[*Price information: one of four versions randomly shown:*]
[*v_p_pays0***]** Now, suppose that the vaccine will be free for the person who wants to be vaccinated.
[*v_p_gets70***]** Now, suppose that the vaccine will be free for a person who wants to be vaccinated, and as an incentive for mass vaccination, the government will pay everyone who wants to be vaccinated 70 zł.
[*v_p_pays10***]** Now, suppose that the vaccine will be fee-based and will cost about 10 zł per person.
[*v_p_pays70***]** Now assume that the vaccine will be fee-based and will cost about 70 zł per person**.**

If, provided the information you just read was confirmed, would you be willing to get vaccinated**?**

definitely not
probably not
probably yes
definitely yes

[P38] Describe below the main reasons for your decision regarding coronavirus vaccination.

[P39] Who or what could change your decision regarding coronavirus vaccination?

[if previously indicated that they would probably or definitely not want to be vaccinated]
[P40] If the vaccine was confirmed to be effective and safe after the first few months of vaccinations, would you be willing to be vaccinated?

definitely not
probably not
probably yes
definitely yes

[P20] To what extent are you concerned or frightened with the current coronavirus pandemic?

[horizontal scale: 1 I am not concerned at all, 10 I am terrified]

[P21] What factors are major influences on the extent to which you are concerned about a coronavirus pandemic?

[P22] How would you rate your impact on whether you get infected with coronavirus?

[horizontal scale 1-7: 1 I have no influence, 7 I have a very high influence]

[P23] How well informed do you feel about the current coronavirus pandemic?

[horizontal scale 1-7: 1 I do not feel informed at all, 7 I feel very well informed]

[Rotate the order of P24 and P25 and register order]
[P24] As of January 14th [wave 1]/February 27th [wave 2] of this year, approximately 1.4 [wave 1]/ 1.68 [wave 2] million people in Poland were confirmed to be infected with the coronavirus.

How many NEW infections do you think will be confirmed in Poland within the next 12 months?

[. . . .] **mln.** people

[P25] As of January 14th [wave 1]/February 27th [wave 2] of this year, approximately 32 [wave 1]/ 43 [wave 2] 000 people in Poland have died as a result of coronavirus infection.

How many people do you think will die from COVID in Poland within the next 12 months?

[. . . .] **thousand** people

[mask] Do you wear a mask when walking down an uncrowded sidewalk?

I never do
I sometimes do
I mostly do
I do every time

[hands] [only wave 2] How often do you wash, disinfect your hands (compared to before the pandemic)?

not more often than before the pandemic
a little more often
a lot more often

[P30] To what extent do you try to keep a physical distance from other people?

[horizontal scale 1 - 10: 1 I do not try to keep my distance at all, 10 I try as much as possible]

[P28] Would you like to add a comment or supplement your above answer (e.g., the reason for the change in a given matter)?

[rotate]
no
yes

[if yes]
[P29] Your comment:

Read the description below and then answer the related questions.

[conspiracy] Certain events, such as the COVID-19 pandemic, are subject to debate. Some people suggest that the official version of these events could be an attempt to hide the truth from the public. This official version could be covering up that these events were planned and covertly prepared by a secret alliance of influential people or organizations (for example, the secret service or the government). We are interested in your opinion on this subject.

To what extent do you agree or disagree with the following statements?

[scale 1-7: 1 strongly disagree, 7 strongly agree]

[rotate and register order]
[r1] I think that official government information on COVID-19 is generally untrue.
[r2] I think that government statistics on COVID-19 infections and deaths are deliberately falsified.
[r3] I think that most of the recommendations related to COVID-19 have no rationale for pandemic containment and actually serve other purposes.

[P31] Are you or have you been infected with coronavirus?

Yes, and this was confirmed by a test
I think so
I don't think so
No

[if in P31: Yes, and this was confirmed by a test]
[P32] Have you been hospitalized due to coronavirus infection?

no
yes

[P33] Do you personally know anyone who has been infected with coronavirus?

no
yes

[if in P33: yes]
[P34] Have any of these people been hospitalized?

no
yes

We're changing the topic.

[M8] Which of the following terms best describes your household?

We live very poorly – we don’t have enough for our basic needs
We live modestly – we have to manage economically every day
We live on average – we have enough money for everyday living, but we have to save for major purchases

We live well – we can afford much without saving
We live very well – we can afford some luxury

[M9] How would you rate your overall health?
very bad
bad
average
good
very good

[M9_1] [only wave 2] To your knowledge, does your health and medical history indicate a particularly high risk of side effects after receiving the COVID-19 vaccination? Such risk factors include certain chronic diseases and allergies.
Yes, I have particular risk factors
No, I do not have any
Don't know

[M9_2] [only wave 2] To your knowledge, does your health and medical history indicate a particularly high risk of severe course of illness if infected with coronavirus? Such risk factors include, but are not limited to, certain chronic diseases, a weakened immune system, cancer, being overweight, diabetes, pregnancy, and smoking.
Yes, I have specific risk factors
No, I do not have any
Don't know

[M9_3] [only wave 2] Do you smoke cigarettes?
yes
no

[M9_3a] If yes, how many cigarettes on average do you smoke per day?

[M9_3b] If no, have you ever smoked cigarettes in your life?
yes
no

[M10] Which description fits you best?
Not a believer
Believer
Believing deeply

[M11] How often do you privately engage in religious activities, for example, prayer, Bible reading, etc.?
Less than once a year
Several times a year
Several times a month
Several times a week
Several times a day

[M12] What is your current employment status?
I work under an employment contract
I work under a contract of commission
I am working on a task-specific contract
I have my own business
Unemployed
Pensioner
Pupil or student

[M13] Did you participate in the Polish Parliament elections held on October 13th, 2019?

no
yes
I don't remember

[if M13: yes]
[M14] Which political group did you vote for in the Polish parliamentary elections that took place on October 13th, 2019?

[rotate]
Prawo i Sprawiedliwość + Solidarna Polska + Porozumienie
Koalicja Obywatelska (PO + Nowoczesna + Inicjatywa Polska + Zieloni)
SLD + Wiosna Roberta Biedronia + Lewica Razem, czyli Partia Razem, Unia Pracy, RSS
PSL + Kukiz 15
Konfederacja (KORWiN + Ruch Narodowy)
other (enter which one) [do not rotate]
I don't remember [do not rotate]

[everyone]
[M15] Did you participate in the 1st round of the presidential election held on June 28th, 2020?

[rotate]
no
yes
I don't remember [do not rotate]

[if yes]
[M16] Who did you vote for in the 1st round of the presidential election held on June 28th, 2020?

[rotate]
Andrzej Duda
Szymon Hołownia
Władysław Kosiniak-Kamysz
Krzysztof Bosak
Rafał Trzaskowski
Robert Biedroń
Marek Jakubiak
Paweł Tanajno
Stanisław Żółtek
Mirosław Piotrowski
Waldemar Witkowski
I don't remember [do not rotate]

[everyone]
[M17] Did you participate in the 2nd round of the presidential election on July 12th, 2020?

[rotate]
no
yes
I don't remember [do not rotate]

[if participated in the 2nd round]
[M18] Who did you vote for in the 2nd round of the presidential election held on July 12th, 2020?

[rotate]
Andrzej Duda

Rafał Trzaskowski
I don’t remember [do not rotate]

[everyone]
[M19] Would you participate in Polish parliamentary elections if they were held this Sunday and participation in them was safe from the perspective of epidemic risk?
definitely not
probably not
probably yes
definitely yes
hard to say

[if rather or definitely yes]
[M20] For which political group would you vote if the elections to the Polish parliament were held this Sunday?
[rotate]
Prawo i Sprawiedliwość + Solidarna Polska + Porozumienie
Koalicja Obywatelska (PO + Nowoczesna + Inicjatywa Polska + Zieloni)
Polska 2050 Szymona Hołowni
SLD + Wiosna Roberta Biedronia + Lewica Razem, czyli Partia Razem, Unia Pracy, RSS
PSL - Koalicja Polska
Kukiz’15
Konfederacja (KORWiN + Ruch Narodowy)
other (enter which one) [do not rotate]
hard to say [do not rotate]

Official and reliable information about the new coronavirus SARS-Cov-2 causing COVID-19 disease can be found, among others, at the following sites in Polish:
www.gov.pl/web/koronawirus, www.pacjent.gov.pl/koronawirusinformacje, www.gis.gov.pl/kategoria/aktualnosci, www.nfz.gov.pl
and English: https://www.who.int/health-topics/coronavirus.
[standard end page with acknowledgments, etc.]

Supplementary S2– Overview: categorization of open-ended questions

We seek further insight into respondents' decisions by analyzing their answers to our open-ended question – "why will you/will you not get vaccinated" and "who or what might change your mind?"; see Supplementary S3 for details of the classification procedure and Supplementary S4 for tables with the prevalence of each response category.

Overall, the most common concern is that of vaccine safety (32% of responses fit into this category), possibly because they have been insufficiently tested (15.9% of responses). It can also be noted that vaccine safety concerns only *grew* from Wave 1 to Wave 2, possibly because of the media reports of the cases of blood clotting disorder in AstraZeneca vaccine patients and consequent decisions of some governments to suspend administration. Such news was indeed most numerous immediately prior to and during Wave 2.

There are also sizable groups just saying no (15.7%) or reporting mistrust towards those producing, distributing, or recommending COVID-19 vaccines (11.5%). The fraction of choice justifications relating to conspiracy theories was relatively low overall (5.4%), even though, when asked explicitly, as many as an approximate 40 percent strongly agree (6 or 7 on a 0-7 scale) that official government information on COVID-19 is generally untrue, that government statistics on COVID-19 infections and deaths are deliberately falsified, and that most of the recommendations related to COVID-19 have no rationale for pandemic containment and actually serve other purposes. Interestingly (though perhaps unsurprisingly), those responses categorized as manifestations of conspiracy theories were nearly four times more prevalent among those saying "definitely not" than those saying they would "probably not" be vaccinated. Likewise, the fraction of those saying they oppose vaccines, in general, was very low (2.2%).

Given the concern about side effects, it is not surprising that among those opposing COVID-19 vaccines, the most common "constructive" responses to the question who or what could change their mind are those mentioning more evidence of safety (6.4%) or more information in general (5.8%) (for more details, see Table S2.2 in Supplementary S4). However, these figures are dwarfed by the prevalence of those unwilling to change one's opinion no matter what (51.7%). Unsurprisingly, this statement was much more common among those who will "definitely" not get vaccinated. Relatedly, when asked if they could change their opinion if the early phase of the vaccination campaign confirmed that the COVID vaccines are safe and effective, as much as 86% of vaccine-opposing respondents said no. Selected significant effects are illustrated in Figure S1.1.

Among respondents willing to get vaccinated, (for more information, see Table S2.3 in Supplementary S4), safety was also of paramount importance, but, obviously, they predominantly claimed that the vaccines will provide protection from the virus for themselves (65%) and/or their loved ones (16.7%). Other popular reasons were convenience of travel and similar concerns (12.3%) and a somewhat diffuse "return to normality" (8.2%). Not surprisingly, those concerned about safety, when asked what could change their mind (Table S2.4 in Supplementary S4), were relatively likely to mention vaccines side effects (these variables correlate at $r = .21$) but, again, overall prevalence even of this most common specific category (22.3%) was much lower than that of the "no-one"/"nothing" kind of responses (44.0%). Selected significant effects are illustrated in Figure S1.2.

Supplementary S3– Procedure: categorization of open-ended questions

The open-ended questions were categorized using the following procedure. Firstly, a number of categories were proposed based on the existing literature and a manual inspection of a sample from the first wave. Initially, the two raters manually categorized the responses of 200 randomly selected subjects, see Table S1.1.

Thus, according to (1), all but one of our kappas could be considered "almost perfect". Subsequently, the process of assigning the responses to categories was partly automatized for the sake of efficiency and objectivity. Specifically, a number of keywords was proposed for each category for the question concerning the main reasons behind the decision ("why?") and the question as to who or what could change the respondent's decision ("who?"), see Supplementary S4. Whenever a keyword was found in the response, we initially assigned the response to the relevant category, allowing a response to belong to multiple categories. For example: category

conspiracy was associated with words (and its inflections) "lie, made up, no pandemics, no COVID, Gates, nonsense". If an algorithm found any of these words (or combinations of words), it classified such responses to the *conspiracy* category. The exact script is available at https://github.com/becarefulwithmath/COVID-classification-of-open-ended-questions. In such a way, our script classified answers to "why?" and "who?". It assigned a preliminary category to 3 716 answers to "why?" question; and to 4 784 answers to "who?" question.

This preliminary categorization was manually inspected by two independent raters and corrections were made if both raters sought them. This was the case for 2 652 initially categorized responses to "why?" and 1 814 of initially categorized responses to "who?" (but many of these changes involved correcting only one of the several categories identified automatically). Moreover, the two raters manually categorized the responses that were not assigned to any category (2 507 of responses to "why?" and 1 439 of responses to "who?"). Again, a response was assigned to a category if both raters agreed.

Supplementary S4– Detailed results: categorization of open-ended questions

This section contains detailed results of the categorization of open questions, meaning Table S2.1, Table S2.2, Table S2.3, and Table S2.4.

Supplementary S5– Additional tables for Wave 1

This section contains additional tables with regression results for Wave 1, in particular Table S3.1, Table S3.2, Table S3.3, and Table S3.4.

Supplementary S6– Additional tables for Wave 2

This section contains additional tables with regression results for Wave 2, in particular Table S4.1, Table S4.2, Table S4.3, and Table S4.4.

Supplementary S7– Summary of demographic characteristics, Wave 1 and Wave 2

This section contains a summary of demographic characteristics for Wave 1 and Wave 2 - Table S5.1

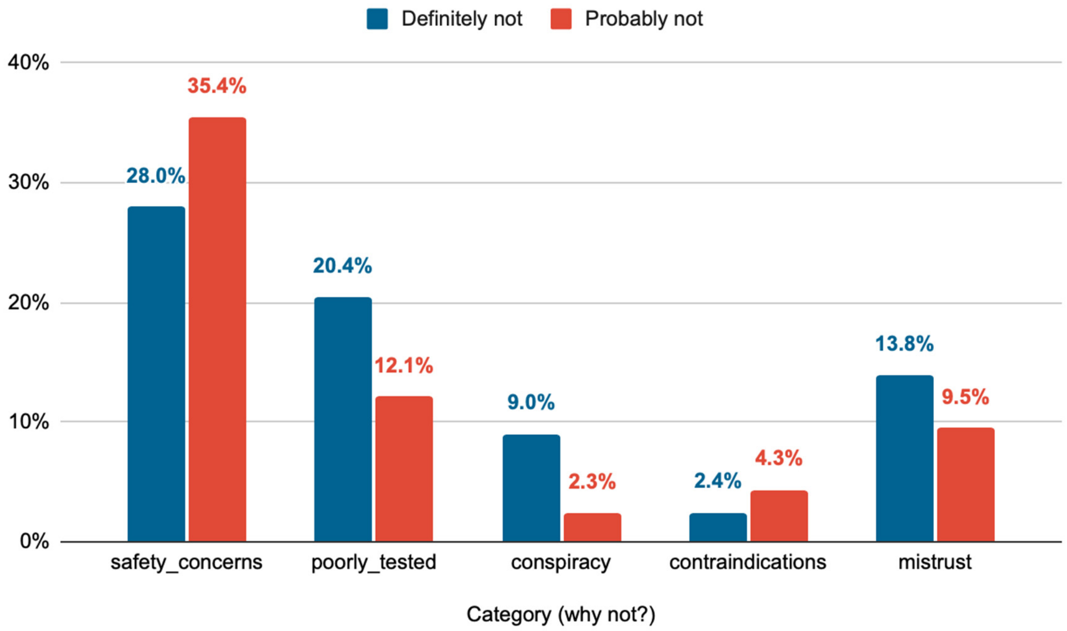

Figure S1.1: Prevalence of the response to the Why question belonging to different categories (definitely not and probably not); weighted average from both waves

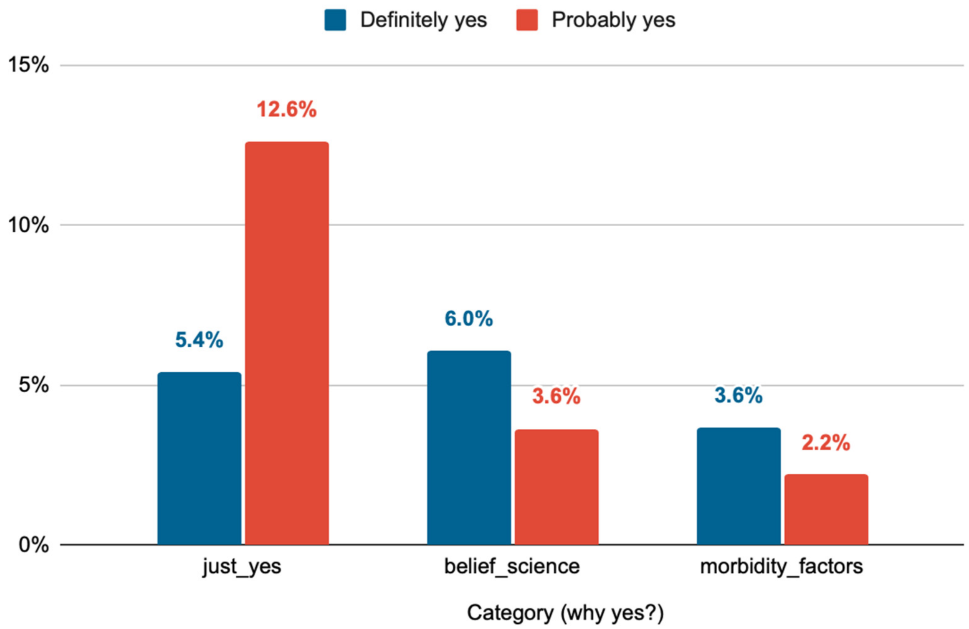

Figure S1.2: Prevalence of the response to the Why question belonging to different categories (definitely yes and probably yes); weighted average from both waves

Table S1.1 - interrater agreement (kappa statistics) for the manual classification of open-ended questions

Variable	Interrater agreement
related to question "why?":	
safety_general	0.98
safety_concerns	0.98
belief_science	0.94
doubts_no	0.83
others_safety	0.97
not_afraid_virus	0.94
poorly_tested	0.96
contraindications	1.00
antibodies	1.00
convenience	0.89
normality	0.83
just_no	1.00
no_alternatives	1.00
just_yes	0.92
conspiracy	1.00
efficacy_concerns	1.00
morbidity_factors	1.00
vaccine_too_costly	1.00
side_effects	0.94
related to question "who?":	
nothing	1.00
doctor	1.00
dont_know	1.00
more_evidence_inefficacy	1.00
else	1.00
forced	1.00
more_evidence_efficacy	0.76
more_evidence_safety	1.00
money	1.00

time	0.85
family	0.94
average	0.96

Two reviewers, n=200

Table S2.1: Categorized answers to: “Why will you not get vaccinated?”

		**Will you get vaccinated?**				
**Why will you not get vaccinated?**		**definitely not**		**probably not**		**Average (weighed by N)**
Classified as:	Examples:	**wave 1**	**wave 2**	**wave 1**	**wave 2**	
safety_concerns	Vaccine unsafe; afraid of complications/of side effects; it's risky; afraid for my health/life	26.4%	29.9%	31.1%	40.6%	32.0%
efficacy_concerns	Vaccine ineffective; I don't know if it will work	5.6%	5.7%	5.1%	7.8%	6.0%
poorly_tested	Poorly tested; lack of accountability by pharmaceutical companies; I will not be a guinea pig	21.4%	19.4%	10.5%	13.9%	15.9%
not_afraid_virus	Virus is not dangerous; I am strong; I don't get sick; I have a high immunity; I am young	10.0%	10.6%	8.2%	7.0%	8.9%
just_no	Just no; I don't want to	18.0%	10.6%	22.2%	9.7%	15.7%
vaccine_too_costly	Vaccine is too costly; I don't have that much money	4.6%	5.8%	5.9%	5.0%	5.4%
conspiracy	Something about conspiracy theories: Gates; 5G; I don't believe in pandemics/COVID/fairy tales	7.9%	10.3%	1.6%	3.2%	5.4%
contraindications	I have medical contraindications (chronic diseases, allergies, sensitivities, chemotherapy)	2.8%	2.0%	5.0%	3.5%	3.4%
antibodies	I've already been through this; I have antibodies	0.5%	1.6%	0.5%	2.4%	1.2%
doubts_no	I have doubts; I don't know what to think about COVID vaccines; I have many unanswered questions	0.9%	0.9%	2.3%	2.4%	1.7%
mistrust_no	I don't trust vaccines/pharmaceutical companies/the government/the media	12.9%	15.0%	7.2%	12.2%	11.5%
antivax	I do not vaccinate; I am against vaccines; vaccines are evil; vaccination is stupid	2.0%	2.6%	2.9%	2.2%	2.4%
	N	*632*	*529*	*745*	*628*	2534
Note: The numbers in Tables 6-9 may add up to more than 100% because some responses belong to more than one category						

Table S2.2: Categorized answers to: “Who or what might change your mind?”

		Will you get vaccinated?				
**Who or what might change your mind?**		**definitely not**		**probably not**		**Average (weighed by N)**
Classified as:	Examples:	**wave 1**	**wave 2**	**wave 1**	**wave 2**	
dont_know	I don't know	8.0%	4.7%	23.2%	13.9%	13.2%
nothing	No one; nothing; extreme (impossible) amounts of money; only me	63.7%	71.1%	35.3%	42.8%	51.7%
family	Close relationships, relatives, family; their health state; if they ended up in hospital	1.6%	2.1%	3.1%	4.8%	2.9%
doctor	Doctor	1.1%	0.4%	2.1%	2.8%	1.7%
else	Some other person/persons mentioned	0.9%	1.5%	2.0%	1.6%	1.5%
more_info	More information about the vaccine; more facts; more studies about the vaccine; better testing of a vaccine	4.1%	3.1%	6.6%	8.9%	5.8%
forced	Force; compulsion; punishment by imprisonment	3.5%	1.4%	3.1%	3.1%	2.8%
money	Money will convince me; if the vaccine is free; it depends on the price	2.8%	3.5%	2.6%	2.5%	2.8%
more_evidence_efficacy	More evidence of effectiveness	1.8%	1.5%	3.6%	3.7%	2.7%
more_evidence_safety	More evidence of safety	4.1%	4.6%	7.8%	8.5%	6.4%
time	I just might decide differently someday; time; years of testing; positive vaccination statistics	4.2%	4.0%	6.6%	4.0%	4.8%
	N	*632*	529	*745*	628	2534

Table S2.3: Categorized answers to: "Why will you get vaccinated?"

		**Will you get vaccinated?**				
**Why will you get vaccinated?**		**definitely yes**		**probably yes**		**Average (weighed by N)**
Classified as:	Examples:	**wave 1**	**wave 2**	**wave 1**	**wave 2**	
safety_general	Less risk; safety; security; more calm; peace of mind; for my own safety; covid is dangerous	60.0%	69.3%	47.3%	61.1%	65.0%
others_safety	For the safety of loved ones; for the safety of others	16.0%	18.8%	12.5%	14.8%	16.7%
normality	A return to normality; I want the pandemic to end; I want this situation to end	7.4%	9.6%	4.8%	6.9%	8.2%
just_yes	Because yes; more pros than cons; it's worth it	7.4%	3.1%	16.6%	8.7%	6.0%
belief_science	Because I believe in science; trust scientists	6.0%	6.1%	3.6%	3.7%	4.9%
no_alternatives	Because there are no alternatives; it is the only option	1.9%	1.7%	1.8%	0.9%	1.3%
morbidity_factors	Individual medical reasons for COVID to be risky (cancer, overweight, diabetes, pregnancy, I'm at risk)	4.9%	2.3%	2.6%	1.8%	2.0%
convenience	To travel; to have more privileges than those not vaccinated	10.6%	12.7%	11.1%	11.9%	12.3%
doubts_yes	I have doubts; I don't know what to think about COVID vaccines; I have many unanswered questions	0.0%	0.2%	2.7%	2.8%	1.5%
money	I don't want to pay; it should be free; I want to make money; I will get vaccinated but for free	0.8%	0.2%	0.7%	1.2%	0.7%
already_vac	Already vaccinated	1.0%	2.6%	0.1%	0.3%	1.4%
obligation	It is necessary; it should be done	1.7%	1.5%	0.9%	1.7%	1.6%
	N	*884*	797	*844*	860	3385

Table S2.4: Categorized answers to: “Who or what might change your mind?”

		Will you get vaccinated?				
**Who or what might change your mind?**		**definitely yes**		**probably yes**		**Average (weighed by N)**
Classified as:	Examples:	**Wave 1**	**Wave 2**	**Wave 1**	**Wave 2**	
dont_know	I don't know	8.2%	5.1%	17.4%	14.6%	11.4%
nothing	No one; nothing; only me	56.0%	54.4%	36.2%	29.7%	44.0%
family	The loved ones, relatives, family; their health state; if they ended up in hospital	2.1%	1.4%	3.3%	7.5%	3.6%
doctor	Doctor	3.1%	4.4%	1.7%	2.6%	2.9%
else	Some other person/persons mentioned	1.0%	1.3%	1.4%	1.3%	1.2%
more_info	New facts about the vaccine	3.7%	1.5%	3.2%	2.2%	2.7%
own_health	Illness; poor health; contraindications	1.5%	1.3%	1.0%	1.8%	1.4%
more_evidence_inefficacy	Evidence of ineffectiveness; negative vaccine test results	6.8%	8.1%	6.7%	10.4%	8.0%
side_effects	Side effects; high mortality; the vaccine will prove harmful	18.5%	21.9%	24.6%	24.1%	22.3%
	N	*884*	797	*844*	860	3385

Table S3.1: Logistic regression on vaccination decision (Wave 1), specifications 1-4

Variable	l_1	l_2	l_3	l_4
v_prod_reputation	0.905	0.970	0.973	0.962
v_efficiency	0.985	0.994	1.001	1.248
v_safety	0.994	1.029	1.033	1.662
v_other_want_it	0.951	0.998	1.008	1.684
v_scientific_authority	0.994	1.063	1.061	1.770
v_vax_passport	1.171	1.279*	1.296*	1.957
v_p_gets70	0.739**	0.800	0.802	0.417*
v_p_pays10	0.866	0.829	0.826	0.944
v_p_pays70	0.585***	0.542***	0.540***	0.536
male	1.388***	1.464***	1.661	2.950*
age	1.033***	1.017***	1.018***	1.028
city_population				
small (<20k)	0.788*	0.689**	0.680**	0.714**
medium (20-99k)	0.887	0.836	0.826	0.846
big (100-500k)	1.013	0.834	0.839	0.841
large (>500k)	1.310*	0.993	0.996	0.995
secondary_edu	1.103	1.167	0.996	1.145
higher_edu	1.763***	1.686***	1.716*	1.721
wealth_low	0.782*	0.899	0.906	0.895
wealth_high	1.366***	1.291**	1.292**	1.285**
health_poor	0.602***	0.570***	0.572**	0.566***
health_good	0.677***	0.784**	0.782**	0.776**
tested_pos_covid	2.363	2.563	2.660	2.308
thinks_had_covid	0.798*	0.976	0.973	0.944
covid_hospitalized	0.697	0.721	0.708	0.763
covid_friends	2.009***	1.621***	1.627***	1.641***
religious	0.850	0.847	0.842	0.836
religious_freq				
less than once a year	0.963	0.960	0.977	0.987
few times a year	1.108	1.067	1.060	1.100
few times a month	0.909	0.843	0.847	0.878
few times a week	0.930	0.867	0.868	0.879
few times a day	0.697*	0.566**	0.571**	0.586**
status_unemployed	0.710**	0.792	0.787	0.781
status_pension	1.478***	1.314*	1.315*	1.265
status_student	1.896***	1.721**	1.739**	1.778**
treatment				
cold	0.893	0.948	0.945	0.930
unemployment	0.840	0.865	0.864	0.845
Performance	0.998	1.548	1.556	1.512
voting_short				
Left		1.415*	2.277	1.431*
right (ruling party)		0.575***	0.390**	0.551***

ultra-right		0.177***	0.199**	0.174***
none or other		0.396***	0.397***	0.387***
e_happiness		0.993	0.991	0.992
e_fear		1.011	1.012	1.010
e_anger		1.001	1.001	1.003
e_disgust		1.054*	1.057*	1.051
e_sadness		0.941*	0.939**	0.940**
e_surprise		1.008	1.006	1.009
risk_overall		0.980	0.980	0.981
risk_work		0.985	0.986	0.985
risk_health		1.049**	1.049**	1.051**
worry_covid		1.105***	1.105***	1.107***
control_covid		1.120***	1.121***	1.119***
informed_covid		1.143***	1.142***	1.144***
informed_cold		0.900**	0.902**	0.902**
informed_unempl		0.980	0.977	0.976
conspiracy_score		0.696***	0.694***	0.691***
subj_est_cases_ln		1.036	1.029	1.040
subj_est_death_l		1.030	1.029	1.030
mask_wearing		1.280***	1.287***	1.275***
distancing		1.105***	1.105***	1.108***
regional dummies	NO	YES, sig.	YES, sig.	YES, sig.
infected_y_pc		41.546*	45.486*	58.150**
deceased_y_pc		4.684	13.185	3.321
PL_infected_yester~y		1.000	1.000	1.000
PL_deceased_yester~y		0.997	0.997	0.997
male#c.age			0.997	
interactions: political preference#education	NO	NO	YES, not sig	NO
interactions: experimental vars#demographic vars	NO	NO	NO	YES, not sig
_cons	0.228***	0.150**	0.147**	0.064**
N	*3105*	*3105*	*3105*	*3105*
pseudo r2	0.120	0.259	0.261	0.264

legend: odds ratios reported; * p<0.1;**p<0.05; *** p<0.01

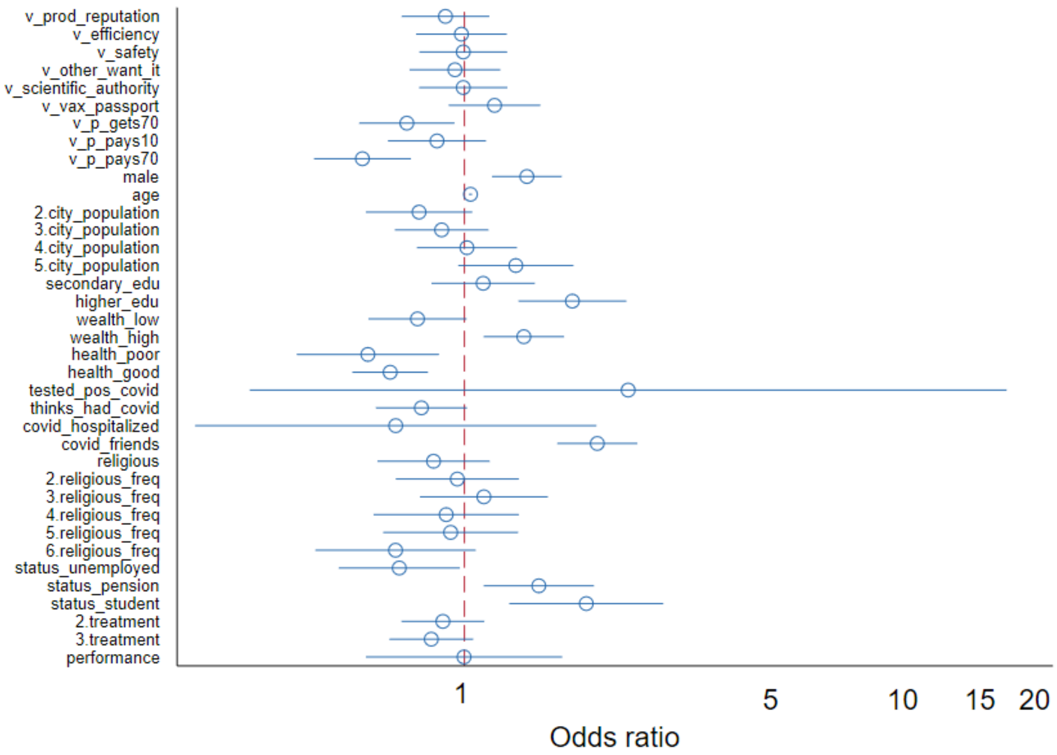

Figure S2.1: Visualization of odds ratio of logistic regression on vaccination decision (Wave 1), specification *l_1* with 95% CIs

Table S3.2: Logistic regression on vaccination decision (Wave 1), specifications 5-8

Variable	l_5	l_6	l_7	l_8
v_prod_reputation	0.511	0.956	0.913	0.958
v_efficiency	0.593	0.983	0.815	0.979
v_safety	0.754	1.024	1.193	0.865
v_other_want_it	0.624	0.989	1.413	1.001
v_scientific_autho~y	0.790	1.064	1.125	1.054
v_vax_passport	0.813	1.264*	1.150	1.284*
v_p_gets70	1.247	0.847	1.266	0.796
v_p_pays10	2.400	0.861	1.627	0.817
v_p_pays70	0.520	0.495***	1.126	0.537***
male	1.467***	1.472***	1.474***	1.468***
age	1.018***	1.017***	1.017***	1.016***
city small (<20k)	0.704**	0.684**	0.676**	0.687**
city medium (20-99k)	0.853	0.836	0.831	0.838
city big (100-500k)	0.839	0.832	0.820	0.839
city large (>500k)	1.010	0.995	0.988	1.007
secondary_edu	1.171	1.190	1.169	1.155
higher_edu	1.669***	1.714***	1.682***	1.693***
wealth_low	0.881	1.045	0.899	0.890
wealth_high	1.268*	1.179	1.300**	1.289**
health_poor	0.548***	0.553***	0.562***	0.573**
health_good	0.769**	0.789**	0.781**	0.782**
tested_pos_covid	2.928	2.684	2.528	2.684
thinks_had_covid	0.993	0.974	0.977	0.990
covid_hospitalized	0.685	0.703	0.725	0.694
covid_friends	1.607***	1.624***	1.629***	1.621***
Religious	0.842	0.845	0.853	0.865
relig:less than once a year	0.948	0.967	0.953	0.954
relig:few times a year	1.071	1.068	1.068	1.043
relig:few times a month	0.819	0.849	0.838	0.832
relig:few times a week	0.884	0.863	0.854	0.842
relig:few times a day	0.567**	0.578**	0.574**	0.556**
status_unemployed	0.814	0.814	0.783	0.802
status_pension	1.321*	1.335*	1.310	1.338*
status_student	1.770**	1.724**	1.716**	1.720**
treatment:cold	0.958	0.939	0.946	0.950
treatment:unemployment	0.857	0.849	0.862	0.855
performance	1.551	1.481	1.551	1.462
voting_short				
left	1.381	1.400	1.402	1.660
right (ruling party)	0.552***	0.577***	0.570***	0.661
ultra-right	0.172***	0.176***	0.173***	0.281***
none or other	0.392***	0.396***	0.394***	0.502***
e_happiness	0.991	0.992	0.993	0.992
e_fear	1.013	1.010	1.009	1.014
e_anger	0.998	1.003	1.003	1.001
e_disgust	1.051	1.055*	1.054	1.056*

e_sadness	0.948*	0.943*	0.943*	0.939**
e_surprise	1.010	1.005	1.006	1.004
risk_overall	0.977	0.978	0.980	0.978
risk_work	0.983	0.985	0.984	0.983
risk_health	1.051**	1.049**	1.048**	1.049**
worry_covid	1.103***	1.106***	1.107***	1.106***
control_covid	1.121***	1.122***	1.123***	1.116***
informed_covid	1.134***	1.142***	1.147***	1.146***
informed_cold	0.903**	0.899**	0.899**	0.902**
informed_unempl	0.988	0.983	0.979	0.983
conspiracy_score	0.691***	0.695***	0.782	0.697***
subj_est_cases_ln	1.040	1.038	1.028	1.032
subj_est_death_l	1.031	1.030	1.030	1.029
mask_wearing	1.284***	1.278***	1.274***	1.287***
distancing	1.110***	1.108***	1.104***	1.105***
regional dummies	YES, sig.	YES, sig.	YES, sig.	YES, sig.
infected_y_pc	42.536*	48.076*	39.511*	37.217*
deceased_y_pc	5.402	6.368	22.937	1.376
PL_infected_yester~y	1.000	1.000	1.000	1.000
PL_deceased_yester~y	0.997	0.997	0.997	0.998
interactions between experimental vars	YES, n.s.	NO	NO	NO
interaction: price#wealth	NO	YES, n.s.	NO	NO
interaction: experimental vars#belief in conspiracy	NO	NO	YES, n.s.	NO
interaction: experimental vars # political preference	NO	NO	NO	YES, n.s.
_cons	0.229	0.147**	0.089*	0.128**
N	*3105*	*3105*	*3105*	*3105*
pseudo *r2*	0.268	0.262	0.261	0.264

legend: odds ratios reported; * p<0.1;**p<0.05; *** p<0.01

Table S3.3: Ordered logistics model (Wave 1), specifications 1-4

Variable	o_1	o_2	o_3	o_4
**v_decision**				
v_prod_reputation	0.880	0.926	0.932	1.075
v_efficiency	0.955	0.911	0.920	1.054
v_safety	0.967	0.968	0.977	1.746*
v_scientific_authority	0.934	0.949	0.963	1.783
v_scientific_autho~y	1.013	1.071	1.077	1.716
v_vax_passport	1.103	1.168	1.181	1.802*
v_p_gets70	0.806**	0.887	0.888	0.500*
v_p_pays10	0.897	0.888	0.880	0.917
v_p_pays70	0.629***	0.589***	0.591***	0.678
male	1.372***	1.428***	1.569*	2.423**
city_population				
small (<20k)	1.019	0.928	0.921	0.962
medium (20-99k)	0.960	0.941	0.934	0.955
big (100-500k)	1.002	0.807	0.815	0.814
large (>500k)	1.448***	1.082	1.087	1.103
secondary_edu	1.084	1.094	0.971	1.084
higher_edu	1.774***	1.550***	1.681*	1.193
wealth_low	0.758**	0.851	0.853	0.845
wealth_high	1.363***	1.311***	1.314***	1.283**
health_poor	0.593***	0.567***	0.570***	0.575***
health_good	0.668***	0.773***	0.773***	0.767***
tested_pos_covid	2.365	4.009	3.982	3.954
thinks_had_covid	0.825**	1.010	1.010	0.972
covid_hospitalized	0.716	0.577	0.585	0.590
covid_friends	1.807***	1.398***	1.401***	1.408***
religious	0.849	0.831	0.835	0.821
religious_freq				
less than once a year	0.963	0.978	0.986	0.994
few times a year	1.029	1.028	1.019	1.044
few times a month	0.927	1.014	1.013	1.045
few times a week	0.904	0.900	0.900	0.910
few times a day	0.727*	0.614**	0.614**	0.635**
status_unemployed	0.759**	0.901	0.899	0.882
status_pension	1.591***	1.316**	1.312**	1.295*
status_student	1.898***	1.647**	1.672**	1.693***
treatment				
cold	0.952	1.032	1.034	1.011
unemployment	0.886	0.922	0.922	0.900
performance	0.875	1.295	1.289	1.284
voting_short				
left		1.480**	1.571	1.486**
right (ruling party)		0.540***	0.464**	0.174***
ultra-right		0.228***	0.282*	0.221***

none or other		0.408***	0.411***	0.396***
e_happiness		0.982	0.982	0.979
e_fear		0.996	0.999	0.993
e_anger		1.003	1.002	1.008
e_disgust		1.012	1.014	1.009
e_sadness		0.974	0.973	0.970
e_surprise		0.999	0.997	1.001
risk_overall		0.994	0.992	1.000
risk_work		0.986	0.987	0.985
risk_health		1.027	1.026	1.026
worry_covid		1.135***	1.135***	1.138***
control_covid		1.113***	1.114***	1.113***
informed_covid		1.132***	1.134***	1.134***
informed_cold		0.935*	0.936*	0.937*
informed_unempl		0.946	0.942	0.946
conspiracy_score		0.673***	0.672***	0.668***
subj_est_cases_ln		1.084	1.084	1.085
subj_est_death_l		1.047**	1.046**	1.048**
mask_wearing		1.241***	1.244***	1.242***
distancing		1.109***	1.109***	1.107***
regional dummies	NO	YES, sig.	YES, sig.	YES, sig.
infected_y_pc		8.165	8.960	9.464
deceased_y_pc		2.407	6.895	0.918
PL_infected_yester~y		1.000	1.000	1.000
PL_deceased_yester~y		0.998	0.998	0.998
male#c.age			0.998	
interactions: political preference#education	NO	NO	YES, n.s.	NO
interactions: experimental vars#demographic vars	NO	NO	NO	YES, n.s.
i_v_p_pays10_male				0.617**
i_v_p_pays70_male				0.603**
cut1	1.050	0.622	0.668	1.526
cut2	4.220***	3.453*	3.719*	8.579**
cut3	16.790***	18.362***	19.880***	46.187***
N	*3105*	*3105*	*3105*	*3105*
pseudo *r2*	*0.080*	*0.185*	*0.186*	*0.189*

legend: * p<0.1;**p<0.05; *** p<0.01

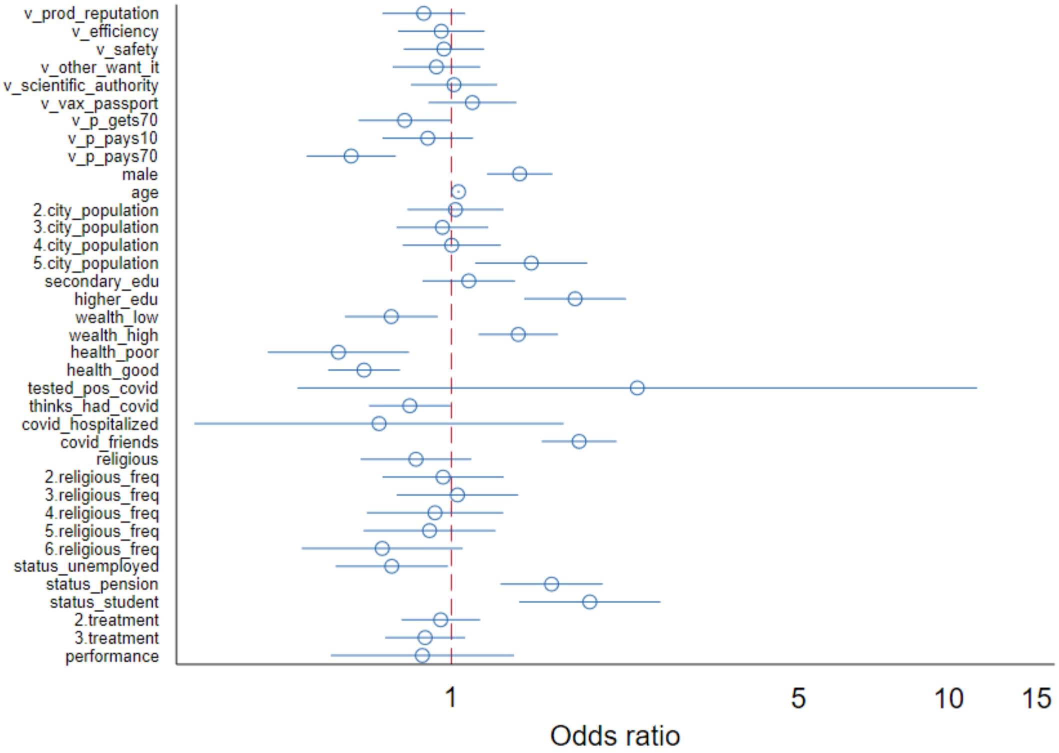

Figure S2.2: Visualization of odds ratio of logistic regression on vaccination decision (Wave 1), specification *o_1* with 95% CIs

Table S3.4: Ordered logistics model (Wave 1), specifications 5-8

Variable	o_5	o_6	o_7	o_8
**v_decision**				
v_prod_reputation	0.814	0.912	0.654	0.863
v_efficiency	0.477	0.902	0.887	0.903
v_safety	0.641	0.960	1.184	1.002
v_other_want_it	0.713	0.943	1.318	0.943
v_scientific_autho~y	0.877	1.066	1.391	1.065
v_vax_passport	0.943	1.159	1.040	1.167
v_p_gets70	1.237	0.833	1.633	0.880
v_p_pays10	1.811	0.871	1.517	0.876
v_p_pays70	0.768	0.484***	1.025	0.582***
male	1.433***	1.432***	1.430***	1.444***
age	1.019***	1.018***	1.018***	1.018***
city small (<20k)	0.930	0.926	0.917	0.932
city medium (20-99k)	0.942	0.946	0.942	0.944
city big (100-500k)	0.798*	0.803*	0.793*	0.813
city large (>500k)	1.084	1.083	1.060	1.090
secondary_edu	1.115	1.120	1.093	1.080
higher_edu	1.557***	1.576***	1.547***	1.545***
wealth_low	0.824	0.830	0.850	0.845

wealth_high	1.310***	1.018	1.320***	1.322***
health_poor	0.565***	0.554***	0.558***	0.570***
health_good	0.773***	0.777***	0.770***	0.772***
tested_pos_covid	3.695	4.376	3.835	4.004
thinks_had_covid	1.006	1.003	1.011	1.011
covid_hospitalized	0.609	0.548	0.589	0.572
covid_friends	1.368***	1.406***	1.409***	1.385***
religious	0.821	0.833	0.842	0.834
relig:less than once a year	0.953	0.978	0.967	0.974
relig:few times a year	1.017	1.022	1.020	1.031
relig:few times a month	1.001	1.021	0.997	1.008
relig:few times a week	0.898	0.892	0.879	0.895
relig:few times a day	0.609**	0.623**	0.624**	0.610**
status_unemployed	0.924	0.904	0.897	0.905
status_pension	1.365**	1.337**	1.323**	1.333**
status_student	1.652**	1.660**	1.665**	1.658**
treatment:cold	1.052	1.027	1.025	1.032
treatment:unemployment	0.923	0.913	0.916	0.924
performance	1.286	1.260	1.294	1.277
voting_short				
left	1.441**	1.477**	1.466**	1.866**
right (ruling party)	0.529***	0.541***	0.538***	0.663*
ultra-right	0.223***	0.229***	0.224***	0.296***
none or other	0.404***	0.410***	0.407***	0.490***
e_happiness	0.981	0.982	0.981	0.983
e_fear	0.994	0.996	0.996	0.996
e_anger	1.001	1.003	1.004	1.004
e_disgust	1.010	1.014	1.011	1.013
e_sadness	0.980	0.976	0.974	0.975
e_surprise	1.001	0.996	0.997	0.997
risk_overall	0.990	0.992	0.991	0.992
risk_work	0.988	0.985	0.988	0.985
risk_health	1.028	1.027	1.027	1.025
worry_covid	1.139***	1.134***	1.138***	1.136***
control_covid	1.113***	1.116***	1.117***	1.110***
informed_covid	1.123***	1.131***	1.132***	1.131***
informed_cold	0.940	0.935*	0.935*	0.937*
informed_unempl	0.949	0.948	0.945	0.949
conspiracy_score	0.669***	0.672***	0.756*	0.672***
subj_est_cases_ln	1.087	1.087	1.077	1.078
subj_est_death_l	1.043**	1.046**	1.047**	1.049**
mask_wearing	1.245***	1.238***	1.236***	1.244***
distancing	1.108***	1.111***	1.106***	1.109***
regional dummies	YES, sig.	YES, sig.	YES, sig.	YES, sig.

infected_y_pc	7.114	9.365	8.019	8.446
deceased_y_pc	1.041	2.791	12.907	0.335
PL_infected_yester~y	1.000	1.000	1.000	1.000
PL_deceased_yester~y	0.998	0.998	0.998	0.998
interaction between experimental vars	YES, n.s.	NO	NO	NO
interaction: price#wealth	NO	YES, n.s.	NO	NO
wp_v_p_pays70_weal~h		1.768**		
interaction: experimental vars#belief in conspiracy	NO	NO	YES, n.s.	NO
interaction: experimental vars # political preference	NO	NO	NO	YES, n.s.
cut1	0.441	0.571	1.044	0.739
cut2	2.471	3.182*	5.816*	4.124**
cut3	13.333***	16.996***	30.996***	22.079***
N	*3105*	*3105*	*3105*	*3105*
pseudo *r2*	*0.189*	*0.187*	*0.186*	*0.187*

legend: * p<0.1;**p<0.05; *** p<0.01

Table S4.1: Logistic regression on vaccination decision (Wave 2), specifications 1-4

Variable	l_1	l_2	l_3	l_4
v_prod_reputation	1.041	1.053	1.042	1.469
v_efficiency	1.035	0.980	0.978	1.165
v_safety	1.047	1.107	1.110	1.099
v_other_want_it	1.039	0.902	0.902	0.809
v_scientific_authority	1.109	1.177	1.166	1.016
v_vax_passport	1.008	0.941	0.941	1.365
v_tested	1.172*	1.031	1.028	2.223**
v_p_gets70	1.122	1.082	1.060	0.923
v_p_pays10	0.997	1.009	1.005	0.819
v_p_pays70	0.748**	0.670**	0.659**	0.934
male	1.482***	1.973***	1.507	1.613
age	1.044***	1.027***	1.025***	1.046***
city_population				
small (<20k)	1.147	1.427*	1.435*	1.460**
medium (20-99k)	1.283*	1.286	1.305	1.264
big (100-500k)	1.296*	1.545***	1.570***	1.485**
large (>500k)	1.151	1.000	1.026	0.994
secondary_edu	1.283*	1.156	1.492	1.179
higher_edu	1.549***	1.189	1.808*	1.080
wealth_low	0.652***	0.817	0.822	0.788
wealth_high	1.247*	1.230	1.213	1.232
health_poor	0.798	0.651*	0.642*	0.636*
health_good	0.641***	0.766*	0.771*	0.766*
vaccine_extra_risky	0.503***	0.515***	0.515***	0.509***
covid_extra_risky	1.472***	1.230	1.219	1.233
health_smoking_light	0.789	0.759	0.713	0.813
health_smoking_mod~e	0.988	0.993	0.969	1.086
tested_pos_covid	3.382	8.499	9.329	7.632
thinks_had_covid	0.837	1.090	1.096	1.089
covid_hospitalized	0.555	0.324	0.310	0.339
covid_friends	2.520***	1.774***	1.768***	1.816***
religious	0.683**	0.542***	0.546***	0.508***
religious_freq				
less than once a year	1.270	1.375	1.407	1.444*
few times a year	1.217	1.276	1.296	1.327
few times a month	1.507**	1.407	1.423	1.444
few times a week	1.670***	1.649**	1.701**	1.750**
few times a day	0.929	0.925	0.952	0.994
status_unemployed	1.032	0.952	0.944	0.956
status_pension	1.093	0.999	1.006	1.014
status_student	1.695**	1.099	1.119	1.057
voting_short				
left		1.311	0.835	1.312
right (ruling party)		0.915	1.530	0.935

ultra-right		0.428***	0.298	0.438***
none or other		0.551***	0.822	0.550***
e_happiness		0.955	0.956	0.954
e_fear		0.956	0.953	0.950
e_anger		0.997	0.994	0.998
e_disgust		1.037	1.041	1.037
e_sadness		1.027	1.031	1.026
e_surprise		0.980	0.981	0.980
risk_overall		0.966	0.968	0.967
risk_work		1.029	1.028	1.030
risk_health		1.012	1.013	1.012
worry_covid		1.165***	1.165***	1.172***
trust_EU_Y		1.900*	1.906*	2.047**
trust_EU_N		0.835	0.837	0.819
trust_gov_Y		0.669	0.677	0.646
trust_gov_N		0.760*	0.753*	0.770
trust_neigh_Y		0.828	0.837	0.780
trust_neigh_N		1.218	1.222	1.238
trust_doctors_Y		1.439*	1.423*	1.397
trust_doctors_N		0.537***	0.534***	0.523***
trust_media_Y		4.173***	4.102***	3.641**
trust_media_N		0.913	0.920	0.920
trust_family_Y		0.990	1.009	0.968
trust_family_N		1.011	1.023	1.007
trust_science_Y		1.718***	1.705***	1.831***
trust_science_N		0.815	0.813	0.844
control_covid		1.118***	1.122***	1.119***
informed_covid		1.133***	1.138***	1.144***
informed_cold		0.975	0.968	0.979
informed_unempl		0.953	0.951	0.950
conspiracy_score		0.708***	0.705***	0.701***
subj_est_cases_ln		0.952	0.951	0.951
subj_est_death_l		1.027	1.026	1.028
mask_wearing		1.179**	1.162**	1.166**
distancing		1.110***	1.110***	1.114***
regional dummies	NO	YES, sig.	YES, sig.	YES, sig.
infected_y_pc		0.827	0.808	0.846
deceased_y_pc		182.399	752.182	57.031
PL_infected_yester~y		1.000	1.000	1.000
PL_deceased_yester~y		1.001	1.001	1.001
male#c.age			1.006	
interactions: political preference#education	NO	NO	YES, n.s.	NO
interactions: experimental vars#demographic vars	NO	NO	NO	YES, n.s.

i_v_tested_age				0.985**
i_v_p_gets70_male				2.416***
_cons	0.058***	0.140***	0.119***	0.062***
N	*2814*	*2814*	*2814*	*2814*
pseudo r2	0.143	0.348	0.350	0.357

legend: odds ratios reported; * p<0.1;**p<0.05; *** p<0.01

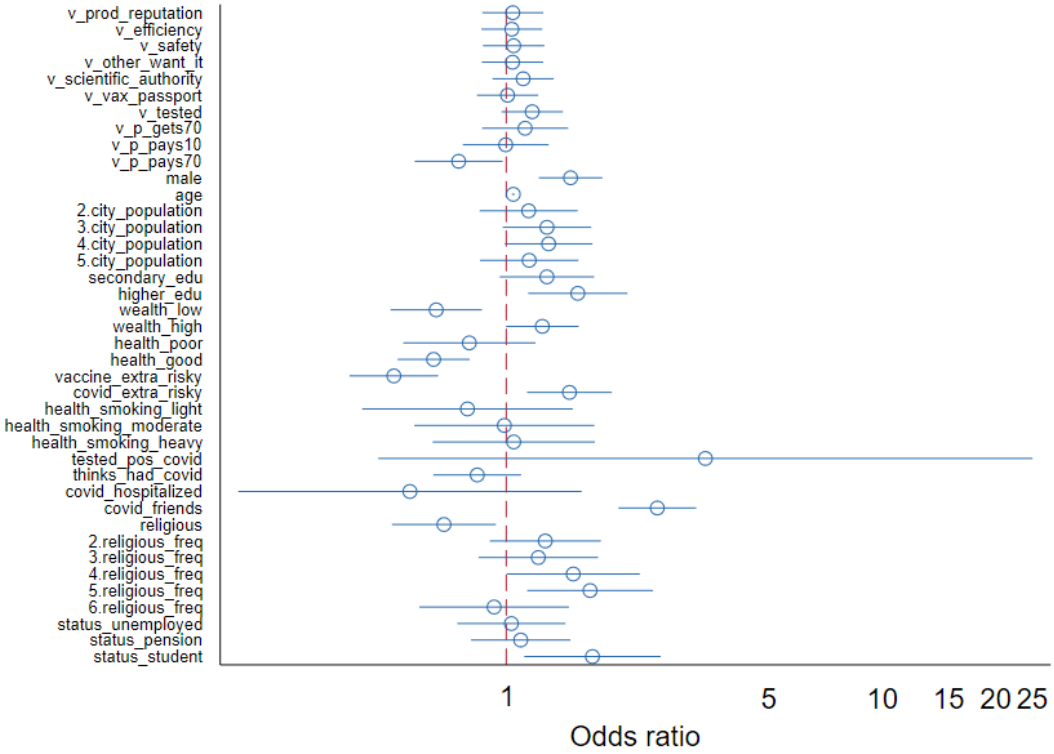

Figure S3.1: Visualization of odds ratio of logistic regression on vaccination decision (Wave 2), specification *l_1* with 95% CIs

Table S4.2: Logistic regression on vaccination decision (Wave 2), specifications 5-9

Variable	l_5	l_6	l_7	l_8	l_9
v_prod_reputation	1.448	1.051	0.328***	1.010	1.096
v_efficiency	1.180	0.979	0.713	0.981	0.757
v_safety	0.843	1.109	1.624	1.276	1.134
v_other_want_it	1.024	0.916	1.098	0.875	1.060
v_scientific_autho~y	0.828	1.179	1.404	1.204	1.081
v_vax_passport	1.146	0.949	1.267	0.924	1.249
v_scientific_authority	0.610	1.033	0.722	1.050	1.495
v_p_gets70	2.273*	1.073	0.469	1.064	1.084
v_p_pays10	0.831	0.950	2.530	0.972	1.038
v_p_pays70	0.454*	0.553***	0.589	0.647***	0.688**
male	1.998***	1.993***	1.975***	1.966***	1.999***
age	1.027***	1.028***	1.029***	1.027***	1.027***
city small (<20k)	1.455**	1.415*	1.482**	1.385*	1.394*
city medium (20-99k)	1.337*	1.281	1.284	1.266	1.252
city big (100-500k)	1.584***	1.541***	1.591***	1.537**	1.551***
city large (>500k)	1.006	0.991	0.988	0.964	0.967
secondary_edu	1.189	1.157	1.157	1.165	1.175
higher_edu	1.233	1.203	1.204	1.170	1.196

wealth_low	0.827	0.720	0.796	0.816	0.814
wealth_high	1.225	1.055	1.221	1.248	1.215
health_poor	0.633*	0.653*	0.660	0.661	0.669
health_good	0.774*	0.762*	0.778*	0.753**	0.765*
vaccine_extra_risky	0.492***	0.508***	0.519***	0.517***	0.513***
covid_extra_risky	1.271	1.228	1.243	1.192	1.226
health_smoking_light	0.723	0.763	0.775	0.734	0.802
health_smoking_mod ~e	0.984	0.995	1.021	0.992	1.046
health_smoking_heav y	1.010	1.061	1.094	1.088	1.119
tested_pos_covid	6.376	8.077	12.247*	8.307	8.136
thinks_had_covid	1.118	1.095	1.119	1.083	1.098
covid_hospitalized	0.380	0.330	0.265*	0.319	0.332
covid_friends	1.783***	1.769***	1.774***	1.813***	1.789***
religious	0.545***	0.549***	0.546***	0.570***	0.525***
relig:less than once a year	1.365	1.354	1.361	1.265	1.437*
relig:few times a year	1.211	1.248	1.274	1.215	1.312
relig:few times a month	1.337	1.396	1.389	1.328	1.437
relig:few times a week	1.583*	1.616**	1.677**	1.552*	1.722**
relig:few times a day	0.878	0.916	0.913	0.847	0.915
status_unemployed	0.943	0.972	0.964	0.983	0.982
status_pension	1.032	0.999	0.994	0.984	1.029
status_student	1.167	1.085	1.115	1.027	1.097
voting_short					
left	1.355	1.320	1.347	1.466	1.281
right (ruling party)	0.921	0.909	0.964	1.230	0.914
ultra-right	0.421***	0.428***	0.426***	0.547	0.416***
none or other	0.564***	0.545***	0.577***	0.509**	0.548***
e_happiness	0.950*	0.955	0.952*	0.957	0.953*
e_fear	0.955	0.957	0.959	0.958	0.96
e_anger	1.006	0.996	0.997	0.998	0.997
e_disgust	1.022	1.037	1.040	1.045	1.034
e_sadness	1.032	1.026	1.020	1.022	1.026
e_surprise	0.990	0.981	0.979	0.978	0.981
risk_overall	0.959	0.966	0.968	0.963	0.968
risk_work	1.028	1.029	1.029	1.029	1.027
risk_health	1.013	1.012	1.015	1.014	1.014
worry_covid	1.165***	1.163***	1.166***	1.165***	1.163***
trust_EU_Y	1.987**	1.950**	1.901*	1.939*	1.935**
trust_EU_N	0.829	0.829	0.867	0.845	0.832
trust_gov_Y	0.636	0.673	0.614	0.667	0.669
trust_gov_N	0.728*	0.757*	0.780	0.739*	0.757*
trust_neigh_Y	0.843	0.833	0.865	0.830	0.831
trust_neigh_N	1.252	1.226	1.180	1.226	1.236

trust_doctors_Y	1.361	1.467*	1.422*	1.444*	1.454*
trust_doctors_N	0.559***	0.536***	0.549***	0.555***	0.536***
trust_media_Y	5.014***	4.342***	4.352***	4.160***	3.864**
trust_media_N	0.931	0.913	0.876	0.174***	0.928
trust_family_Y	1.048	0.985	0.988	0.967	1.000
trust_family_N	1.001	1.013	1.037	1.052	1.017
trust_science_Y	1.761***	1.701***	1.773***	1.671***	1.724***
trust_science_N	0.799	0.819	0.789	0.807	0.828
control_covid	1.117***	1.118***	1.118***	1.123***	1.118***
informed_covid	1.141***	1.141***	1.129***	1.136***	1.138***
informed_cold	0.960	0.973	0.978	0.971	0.977
informed_unempl	0.964	0.949	0.956	0.945	0.953
conspiracy_score	0.696***	0.707***	0.631***	0.703***	0.704***
subj_est_cases_ln	0.955	0.955	0.961	0.956	0.947
subj_est_death_l	1.021	1.026	1.032	1.029	1.028
mask_wearing	1.184**	1.181**	1.191***	1.171**	1.180**
distancing	1.113***	1.109***	1.108***	1.109***	1.114***
regional dummies	YES, sig.	YES, sig.	YES, sig.	YES, sig.	YES, sig.
infected_y_pc	0.828	0.828	0.787	0.838	0.797
deceased_y_pc	35.555	213.322	547.450	185.755	326.051
PL_infected_yester~y	1.000	1.000	1.000	1.000	1.000
PL_deceased_yester~y	1.001	1.001	1.001	1.001	1.001
interactions between experimental vars	YES, n.s.	NO	NO	NO	NO
interaction: price#wealth	NO	YES, n.s.	NO	NO	NO
interaction: experimental vars#belief in conspiracy	NO	NO	YES, n.s.	NO	NO
v_prod_reputat_con~y			1.282***		
interaction: experimental vars # political preference	NO	NO	NO	YES, n.s.	YES, n.s.
_cons	0.156**	0.146**	0.180*	0.132***	0.212*
N	*2814*	*2814*	*2814*	*2814*	*2814*
pseudo *r2*	*0.363*	*0.350*	*0.356*	*0.356*	*0.352*

legend: odds ratios reported; * p<0.1;**p<0.05; *** p<0.01

Table S4.3: Ordered logistics model (Wave 2), specifications 1-4

Variable	o_1	o_2	o_3	o_4
**v_decision**				
v_prod_reputation	1.086	1.143	1.127	1.224
v_efficiency	1.096	1.059	1.054	1.132
v_safety	1.016	1.088	1.085	1.186
v_other_want_it	1.137	1.023	1.018	0.711
v_scientific_autho~y	1.021	1.038	1.031	1.188
v_vax_passport	1.046	1.027	1.028	1.629
v_tested	1.234***	1.069	1.075	1.551
v_p_gets70	1.056	0.988	0.965	0.906
v_p_pays10	0.982	0.911	0.903	0.782
v_p_pays70	0.724***	0.628***	0.618***	0.840
male	1.413***	1.760***	1.233	1.855**
age	1.043***	1.024***	1.021***	1.032***
city_population				
small (<20k)	1.122	1.335**	1.332**	1.370**
medium (20-99k)	1.124	1.052	1.064	1.030
big (100-500k)	1.203	1.294**	1.324**	1.257*
large (>500k)	1.140	0.981	1.011	0.991
secondary_edu	1.233*	1.118	1.630*	1.125
higher_edu	1.560***	1.188	1.945**	1.264
wealth_low	0.646***	0.856	0.860	0.866
wealth_high	1.273**	1.247**	1.242**	1.244*
health_poor	0.914	0.793	0.780	0.807
health_good	0.712***	0.892	0.897	0.899
vaccine_extra_risky	0.523***	0.597***	0.601***	0.599***
covid_extra_risky	1.602***	1.409***	1.395***	1.410***
health_smoking_light	0.845	0.911	0.851	0.945
health_smoking_mod ~e	1.048	1.040	1.015	1.103
health_smoking_heav y	1.115	1.130	1.094	1.189
thinks_had_covid	0.907	1.172	1.190	1.171
covid_hospitalized	0.649	0.389*	0.379*	0.402*
covid_friends	2.277***	1.515***	1.516***	1.536***
religious	0.717**	0.638***	0.642***	0.614***
religious_freq				
less than once a year	1.202	1.323*	1.338*	1.331*
few times a year	1.127	1.213	1.223	1.236
few times a month	1.188	1.148	1.158	1.175
few times a week	1.349*	1.350*	1.389*	1.382*
few times a day	0.822	0.984	1.017	1.036
status_unemployed	1.058	0.967	0.955	0.960
status_pension	1.160	1.037	1.047	1.057
status_student	1.978***	1.170	1.207	1.166
voting_short				

left		1.112	1.001	1.118
right (ruling party)		0.785	1.325	0.795
ultra-right		0.459***	0.353	0.471***
none or other		0.567***	0.947	0.573***
e_happiness		0.980	0.981	0.979
e_fear		0.954*	0.951*	0.952*
e_anger		0.983	0.981	0.980
e_disgust		1.022	1.022	1.026
e_sadness		1.033	1.039	1.029
e_surprise		0.976	0.979	0.979
risk_overall		0.959	0.961	0.955*
risk_work		1.015	1.014	1.018
risk_health		1.024	1.025	1.024
worry_covid		1.183***	1.183***	1.184***
trust_EU_Y		2.147***	2.135***	2.258***
trust_EU_N		0.794**	0.795**	0.789**
trust_gov_Y		0.585*	0.578*	0.606*
trust_gov_N		0.932	0.916	0.944
trust_neigh_Y		1.102	1.100	1.062
trust_neigh_N		1.171	1.169	1.178
trust_doctors_Y		1.387**	1.381**	1.338*
trust_doctors_N		0.579***	0.576***	0.568***
trust_media_Y		2.134	2.197	2.198
trust_media_N		0.879	0.884	0.867
trust_family_Y		0.861	0.875	0.857
trust_family_N		0.936	0.939	0.960
trust_science_Y		1.839***	1.836***	1.913***
trust_science_N		0.805	0.810	0.829
control_covid		1.099***	1.102***	1.100***
informed_covid		1.059	1.061	1.067*
informed_cold		0.977	0.972	0.976
informed_unempl		0.968	0.968	0.965
conspiracy_score		0.702***	0.699***	0.698***
subj_est_cases_ln		0.961	0.965	0.954
subj_est_death_l		1.021	1.021	1.020
mask_wearing		1.215***	1.198***	1.224***
distancing		1.104***	1.104***	1.104***
regional dummies	NO	YES, sig.	YES, sig.	YES, sig.
infected_y_pc		0.950	0.919	0.982
deceased_y_pc		238342.666	1123990.115*	114008.661
PL_infected_yester~y		1.000	1.000	1.000
PL_deceased_yester~y		1.000	1.000	1.000
male#c.age			1.008	
interactions: political preference#education	NO	NO	YES, n.s.	NO

interactions: experimental vars#demographic vars	NO	NO	NO	YES, n.s.
i_v_vax_passport_m~e				0.651**
cut1	4.320***	1.216	1.467	1.930
cut2	17.048***	7.948***	9.625***	12.771***
cut3	86.588***	68.142***	83.134***	112.167***
N	*2814*	*2814*	*2814*	*2814*
pseudo *r2*	*0.090*	*0.247*	*0.249*	*0.252*

legend: * p<0.1;**p<0.05; ***p<0.01

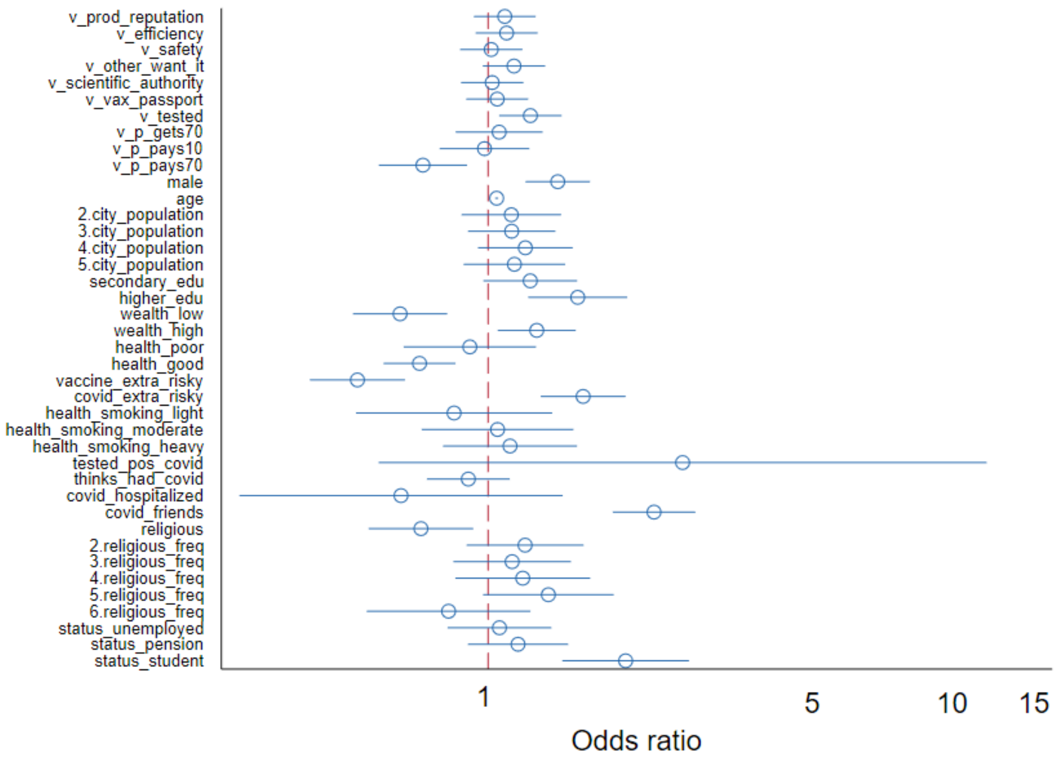

Figure S3.2: Visualization of odds ratio of logistic regression on vaccination decision (Wave 2), specification *o_1* with 95% CIs

Table S4.4: Ordered logistics model (Wave 2), specifications 5-9

Variable	o_5	o_6	o_7	o_8	o_9
**v_decision**					
v_prod_reputation	1.336	1.143	0.714	1.157	1.222
v_efficiency	1.129	1.062	0.963	1.065	0.846
v_safety	0.701	1.086	1.293	1.135	1.150
v_other_want_it	0.990	1.037	1.114	0.997	1.198
v_scientific_autho~y	0.821	1.041	0.953	1.057	1.174
v_vax_passport	1.780**	1.028	0.837	1.020	1.085
v_tested	0.818	1.074	0.787	1.074	1.165
v_p_gets70	1.001	0.906	0.706	0.979	0.982
v_p_pays10	0.825	0.966	1.326	0.892	0.908
v_p_pays70	0.452**	0.518***	0.662	0.616***	0.630***
male	1.735***	1.784***	1.757***	1.749***	1.781***
age	1.025***	1.024***	1.024***	1.024***	1.024***
city small (<20k)	1.331**	1.328**	1.354**	1.311*	1.330**
city medium (20-99k)	1.054	1.054	1.057	1.053	1.045
city big (100-500k)	1.260*	1.298**	1.297**	1.294*	1.311**
city large (>500k)	0.979	0.976	0.976	0.954	0.965
secondary_edu	1.103	1.132	1.128	1.106	1.132
higher_edu	1.185	1.204	1.182	1.171	1.198
wealth_low	0.854	0.647*	0.857	0.861	0.850
wealth_high	1.247**	1.233	1.240**	1.256**	1.235*
health_poor	0.790	0.817	0.805	0.796	0.800
health_good	0.882	0.890	0.902	0.883	0.888
vaccine_extra_risky	0.586***	0.585***	0.597***	0.600***	0.592***
covid_extra_risky	1.424***	1.417***	1.419***	1.378***	1.406***
health_smoking_light	0.946	0.927	0.911	0.935	0.962
health_smoking_mod ~e	1.060	1.045	1.050	1.058	1.076
health_smoking_heav y	1.139	1.152	1.139	1.169	1.172
tested_pos_covid	6.293*	5.797*	7.291*	6.184*	6.592*
thinks_had_covid	1.173	1.173	1.184	1.169	1.179
covid_hospitalized	0.395*	0.408*	0.359*	0.383*	0.385*
religious	0.649***	0.641***	0.633***	0.662***	0.631***
relig:less than once a year	1.321*	1.295	1.325*	1.257	1.340*
relig:few times a year	1.180	1.205	1.213	1.163	1.221
relig:few times a month	1.114	1.145	1.150	1.097	1.154
relig:few times a week	1.325	1.332	1.356*	1.287	1.372*
relig:few times a day	0.933	0.973	0.988	0.928	0.976
status_unemployed	0.967	0.965	0.975	0.999	0.987
status_pension	1.043	1.037	1.052	1.028	1.047
status_student	1.218	1.170	1.169	1.134	1.159
voting_short					
left	1.141	1.130	1.104	1.184	1.106

right (ruling party)	0.783	0.792	0.785	1.015	0.795
ultra-right	0.459***	0.451***	0.455***	0.442**	0.454***
none or other	0.565***	0.565***	0.573***	0.542***	0.567***
e_happiness	0.979	0.982	0.977	0.981	0.980
e_fear	0.956*	0.954*	0.956*	0.955*	0.954*
e_anger	0.983	0.981	0.985	0.986	0.982
e_disgust	1.017	1.023	1.021	1.024	1.020
e_sadness	1.035	1.035	1.028	1.028	1.034
e_surprise	0.976	0.975	0.975	0.976	0.978
risk_overall	0.955*	0.960	0.960	0.957	0.959
risk_work	1.015	1.014	1.016	1.016	1.014
risk_health	1.025	1.023	1.026	1.024	1.025
worry_covid	1.181***	1.182***	1.185***	1.183***	1.182***
trust_EU_Y	2.156***	2.163***	2.128***	2.145***	2.190***
trust_EU_N	0.791**	0.794**	0.797**	0.794**	0.793**
trust_gov_Y	0.621*	0.581*	0.556**	0.583*	0.587*
trust_gov_N	0.929	0.925	0.940	0.921	0.929
trust_neigh_Y	1.094	1.111	1.112	1.121	1.094
trust_neigh_N	1.180	1.185	1.156	1.185	1.191
trust_doctors_Y	1.337*	1.389**	1.393**	1.404**	1.392**
trust_doctors_N	0.593***	0.582***	0.587***	0.584***	0.586***
trust_media_Y	2.309*	2.155	2.153	0.174***	2.095
trust_media_N	0.900	0.872	0.866	0.861	0.891
trust_family_Y	0.879	0.863	0.856	0.841*	0.867
trust_family_N	0.943	0.944	0.959	0.939	0.930
trust_science_Y	1.831***	1.827***	1.883***	1.829***	1.852***
trust_science_N	0.810	0.804	0.794*	0.808	0.807
control_covid	1.099***	1.098***	1.098***	1.098***	1.103***
informed_covid	1.061	1.063	1.053	1.057	1.058
informed_cold	0.975	0.975	0.980	0.975	0.978
informed_unempl	0.967	0.968	0.972	0.965	0.968
conspiracy_score	0.698***	0.701***	0.634***	0.702***	0.702***
subj_est_cases_ln	0.947	0.964	0.965	0.967	0.964
subj_est_death_l	1.017	1.021	1.023	1.023	1.020
mask_wearing	1.216***	1.217***	1.214***	1.212***	1.214***
distancing	1.108***	1.103***	1.104***	1.104***	1.106***
regional dummies	YES, sig.	YES, sig.	YES, sig.	YES, sig.	YES, sig.
infected_y_pc	0.992	0.946	0.929	0.975	0.947
deceased_y_pc	30285.575	423049.653*	448809.409*	209639.191	197672.969
PL_infected_yester~y	1.000	1.000	1.000	1.000	1.000
PL_deceased_yester~y	1.000	1.000	1.000	1.000	1.000
interaction between experimental vars	YES, n.s.	NO	NO	NO	NO
interaction: price#wealth	NO	YES, n.s.	NO	NO	NO

interaction: experimental vars#belief in conspiracy	NO	NO	YES, n.s.	NO	NO
interaction: experimental vars # political preference	NO	NO	NO	YES, n.s.	NO
interactions: experimental var shown#shown as 1st	NO	NO	NO	NO	YES, n.s.
interactions: experimental var shown#order of vars	NO	NO	NO	NO	YES, n.s.
cut1	1.072	1.187	0.797	1.276	1.062
cut2	7.157***	7.792***	5.262**	8.475***	6.985***
cut3	62.839***	67.289***	45.407***	73.573***	60.331***
N	*2814*	*2814*	*2814*	*2814*	*2814*
pseudo *r2*	*0.253*	*0.249*	*0.249*	*0.251*	*0.249*

legend: * p<0.1;**p<0.05; *** p<0.01

Table S5.1 Summary statistics - demographic characteristics, Wave 1 and Wave 2

	Wave 1			Wave 2		
	Mean	Median	SD	Mean	Median	SD
city_population	2.590	3	1.450	2.614	3	1.455
male	0.483	0	0.500	0.482	0	0.500
age	43.718	44	15.889	45.826	46	16.493
elementary_edu	0.129	0	0.335	0.127	0	0.333
secondary_edu	0.439	0	0.496	0.445	0	0.497
higher_edu	0.432	0	0.495	0.429	0	0.495
wealth_low	0.164	0	0.370	0.156	0	0.363
wealth_high	0.285	0	0.451	0.296	0	0.456
health_poor	0.077	0	0.266	0.079	0	0.269
health_good	0.549	1	0.498	0.555	1	0.497
religious_often	0.414	0	0.493	0.399	0	0.490
status_unemployed	0.103	0	0.304	0.106	0	0.308
status_pension	0.211	0	0.408	0.232	0	0.422
status_student	0.062	0	0.242	0.069	0	0.253

SI References

1. J. R. Landis, G. G. Koch, An application of hierarchical kappa-type statistics in the assessment of majority agreement among multiple observers. *Biometrics*, 363–374 (1977).
